# Resistance Mechanisms of *Sitobion miscanthi* (Hemiptera: Aphididae) to Malathion Revealed by Synergist Assay

**DOI:** 10.3390/insects13111043

**Published:** 2022-11-11

**Authors:** Tianyang Xu, Kai Lou, Dunlun Song, Bin Zhu, Pei Liang, Xiwu Gao

**Affiliations:** Department of Entomology, China Agricultural University, Beijing 100193, China

**Keywords:** *S. miscanthi*, malathion resistance, synergists, detoxification enzymes

## Abstract

**Simple Summary:**

The resistance of *Sitobion miscanthi* to malathion possessed low realized heritability. No cross-resistance was found to imidacloprid in the resistant strain. The increase in esterase was involved in *S. miscanthi* resistance to malathion by the assay of inhibitor synergism and esterase activity.

**Abstract:**

A resistant strain (MRS) of *Sitobion miscanthi* was cultured by continuous selection with malathion for over 40 generations. The MRS exhibited 32.7-fold resistance to malathion compared to the susceptible strain (MSS) and 13.5-fold, 2.9-fold and 4.8-fold cross-resistance for omethoate, methomyl and beta-cypermethrin, respectively. However, no cross-resistance was found to imidacloprid in this resistant strain. The realized heritability for malathion resistance was 0.02. Inhibitors of esterase activity, both triphenyl phosphate (TPP) and S,S,S,-tributyl phosphorotrithioate (DEF) as synergists, exhibited significant synergism to malathion in the MRS strain, with 11.77-fold and 5.12-fold synergistic ratios, respectively, while piperonyl butoxide (PBO) and diethyl maleate (DEM) showed no significant synergism in the MRS strain. The biochemical assay indicated that carboxylesterase activity was higher in MRS than in MSS. These results suggest that the increase in esterase activity might play an important role in *S. miscanthi* resistance to malathion. Imidacloprid could be used as an alternative for malathion in the management of wheat aphid resistance.

## 1. Introduction

Wheat aphids are agricultural pests found worldwide, which cause damage directly through feeding, or indirectly by transmitting pathogenic viruses, especial the barley yellow dwarf virus (BYDV) [1,2]. Wheat aphids are ubiquitous and destructive in the main areas of wheat production of China, leading to a 10% reduction in wheat crop production annually [3,4]. The grain aphid, *Sitobion miscanthi* was the dominant species in the middle and late stages of wheat growth in China [5]. The application of chemical insecticides is the main approach for the management of wheat aphids [6,7]. Organophosphorus insecticides have been widely used for aphid control. However, the development of insecticide resistance has resulted in the decrease in control efficacy of aphids. The field population of the grain aphid showed 15-fold resistance to omethoate in 1988 in China [8]. Malathion, as an important organophosphorus insecticide, was widely applied for aphid control in China. Gao et al. (1992) found that the field population of the green peach aphid, *Myzus persicae* Sulzer from Beijing developed 32-fold resistance to malathion in 1991 [9]. The resistant mechanisms of the green peach aphid to malathion were not only related to the increase in carboxylesterase (CarEs) activity, but also related to the increase in the mixed-functional oxidase activity and the decrease in acetylcholinesterase (AChE) sensitivity [9]. The resistance of brown planthopper to malathion was also related to the increase in carboxylesterase activity that played an important role in the early stage of resistance development [10].

The carboxylesterases (CarEs), glutathione *S*-transferase (GSTs) and cytochrome P450 monooxygenase (CYP) are three important types of detoxifying enzymes against insecticides in insect pests [11,12,13,14,15]. The increase in their activity can result in the increase in insect pest resistance to insecticides. DEM, DEF and PBO as inhibitors of GSTs, CarEs and CYP, respectively, are often used in vivo to identify the mechanisms of insect pest resistance to insecticides [16,17].

In this study, we established the resistant strain of the *S. miscanthi* through continuous selection with malathion. Assays of both synergism by inhibitors of esterase and carboxylesterase activity suggested that the increase in esterase activity was involved in malathion resistance of *S. miscanthi*. The assay of cross-resistance indicated that imidacloprid could be used as an alternative for malathion to manage resistance in wheat aphids.

## 2. Materials and Methods

### 2.1. Insects

*S. miscanthi* was cultured on wheat seedlings in chamber of 25 ± 1 °C, 60 ± 10% relative humidity, and a photoperiod of 17:7 h (light: dark), which was collected from Sichuan province.

### 2.2. Chemicals

Malathion (*rac*-diethyl 2-[(dimethoxyphosphorothioyl)sulfanyl] succinate) (97.0%) and methomyl (*S*-methyl-*N*-[(methylcarbamoyl)oxy] thioacetimidate) (98%) were obtained from Tianjin Aigefu Co., Ltd. Imidacloprid (1-[(6-chloro-3-pyridinyl)methyl]-*N*-nitro-2-imidazolidinimine) (95.3%) was purchased from Shijiazhuang Chemical Co., Ltd. Beta-cypermethrin ([(*R*)-cyano-(3-phenoxyphenyl)methyl] (1*R*,3*S*)-3-(2,2-dichloroethenyl)-2,2-dimethylcyclopropane-1-carboxylate) (93%) and omethoate (O,O-dimethyl-S-methylcarbamoylmethylthiophosphate) (40%) were obtained from Hebei Xinxing Chemical Co., Ltd. Sodium hydrogen phosphate (DSP) (98%) and potassium dioxyphosphate (98%) were obtained from Beijing Chemical Reagent Co., Ltd. Bovine serum albumin (BSA) (99%) was purchased from Beijing Tongzheng Biological Co., Ltd. Eserine (99%), α-naphthyl acetate (α-NA) (98%), acetylthiocholine iodide (ATCh) (99%), 5,5′-dithio-bis-2-nitrobenzoic acid (DTNB) (98%), triton X-100 (99%), ethylene diamine tetraacetic acid (EDTA) (99%), 1-chloro-2,4-dinitrobenzene (CDNB) (99%), reduced glutathione (GSH) (98%), fast blue B salt, coomassie brilliant blue G-250 (99%), sodium dodecyl sulfate (SDS) (99%), triphenyl phosphate (TPP) (99%), piperonyl butoxide (PBO) (99%) and diethyl maleate (DEM) (98%) were purchased from Sigma-Aldrich Chemical Reagent Co., Ltd. in America. S, S, S-tributyl phosphorotrithioate (DEF) (98%) was purchased from ChemService (West Chester, PA, USA).

### 2.3. Bioassays

The glass tube film method was used in bioassays. Insecticide was dissolved in acetone. The glass tube (inner surface = 36.0 cm^2^) was rotated in a micro-rotator, with two hundred and fifty microliters of the desired concentration solution or acetone for the control treatment, until the acetone volatilized. Then, 20 apterous adult aphids were transferred to each tube, and each treatment had three replicates. Mortality was scored at three hours after treatment [18].

### 2.4. Selection

The single colony method was used in the backward elimination procedure to obtain a malathion susceptible strain (MSS). Ten aphids, randomly selected from original *S. miscanthi* strain, fed on wheat seedlings, respectively. The offspring of these ten aphids developed ten groups and their susceptibility was determined by LC_50_ dose of malathion in the previous generation. The *S. miscanthi* population with the highest mortality was used as the F_1_ generation, which was under successive selection to produce a susceptible strain.

The resistant strain (MRS) was obtained by the aphid-dipping method with LC_50_ dose of malathion for resistance screening in each generation [19]. Wheat seedlings with aphids were immersed in malathion solution for 20 s. After 24 h, the surviving aphids were cultured as the source for next generation selection for mortality was maintained at 40–80%. Meanwhile, bioassays were performed every 4 generations.

### 2.5. Cross-Resistance Pattern and Synergist Study

Toxicity tests of omethoate, methomyl, imidacloprid and beta-cypermethrin were conducted by the method of glass tube film in MRS and MSS. The pretreatment experiment of synergists was conducted by dipping leaf with aphids (the total number of aphids was about 300–400 individuals) into 0.05% triton X-100 solution containing the desired concentration of synergist (TPP 500 mg L^−1^, PBO 500 mg L^−1^, DEM 700 mg L^−1^ or DEF 700 mg L^−1^) for 20 s. Then, these leaves with aphids were transferred to culture dish (diameter 9 cm) for 24 h. The surviving individuals were used to measure the toxicity of malathion by the method of glass tube film.

### 2.6. Biochemical Assays

#### 2.6.1. Carboxylesterase Activity Assays

Carboxylesterase (CarEs) activity were measured with α-NA as substrate [20]. Thirty apterous adults were homogenized in an ice bath with 1 mL 0.1 M phosphate buffer of pH 7.0. Then, the homogenate was centrifuged at 4 °C for 15 min at 10,800 rpm (ASTEC Microtec 1524R centrifuge, Japan), and the supernatant was collected as the enzyme source. The reaction system contained 1.8 mL α-NA (3 × 10^−4^ M), 0.45 mL phosphate buffer (0.04 M, pH 7.0) and 50 μL enzyme liquid prepared above, incubated at 30 °C for 15 min. Subsequently, 0.9 mL chromogenic reagent, according to the 2:5 proportion of mixing 1% fast blue B salt and 5% SDS, was added to stop the reaction. After standing at room temperature for 15 min, the value of optical density (OD) was measured at 600 nm. Michaelis constants (*K*m) and maximal velocities (*V*max) were measured for α-NA which was diluted serially to 8 concentrations in homogenization buffer, and calculated using Enzfit software (Elservier, Cambridge, UK).

#### 2.6.2. Glutathione S-Transferase Assays

Activities of glutathione *S*-transferase (GSTs) were measured with CDNB as substrate [21]. The preparation of the enzyme source was similar as Section 2.6.1, however, with phosphate buffer (0.1 M, pH 6.5 and containing 1.0 mmol/L EDTA). The reaction system included 30 μL CDNB (30 mM), 30 μL GSH (30 mM), 30 μL enzyme liquid and 810 μL phosphate buffer (0.1 M, pH 6.5 containing 1.0 mmol/L EDTA). After rapid mixing at 25 °C, the change in OD was recorded at 340 nm for 2 min as the reaction rate in an UV/VIS Spectrometer Lambda Bio-40 (Per-kin-Elmer, USA).

#### 2.6.3. Acetylcholinesterase Assays

Activities of acetylcholinesterase (AChE) were measured by the method of Ellman et al. (1961) [22]. The preparation of the enzyme source was similar as Section 2.6.1, however, using phosphate buffer (0.1 M, pH 7.5 and containing 0.1% Triton X-100). The reaction mixture of 0.1 mL ATCh (10 mM) and 0.1 mL enzyme solution was incubated at 30 °C for 15 min. Then, adding 3.6 mL DTNB (containing 40% ethanol) to the reaction mixture, the value of OD was measured at 412 nm.

### 2.7. Protein Assays

The protein of *S. miscanthi* was measured by the method of Bradford (1976) with G-250 as coloring agent, BSA was used as the standard protein [23].

### 2.8. Statistical Analysis

The probit analysis of LC_50_ values based on bioassays was conducted with POLO software (LeOra Software Inc., Cary, NC, USA).

Realized heritability (*h*^2^) was calculated by the following formula: *h^2^ = R/S* [22], in which *R* represented selection effect$\;R=\left[\log\left({\mathrm{final}\;\mathrm{LC}}_{50}\right)-\log\left({\mathrm{initial}\;\mathrm{LC}}_{50}\right)\right]/n$, *S* represent selection differential, *S = i δ_p_* and *n* was the number of selection generations, where *i* presented the intensity of selection and was calculated by the following formula:$\;i=1.583-0.0193336p+0.0000428p^{2}+3.65194/p$. *δ_p_* was the phenotypic standard deviation, counted as $\delta_{p}={\left[1⁄2\left(initial\;slop+final\;slop\right)\;\right]}^{-1}$ [24].

## 3. Results

### 3.1. Selection

**Figure 1:**
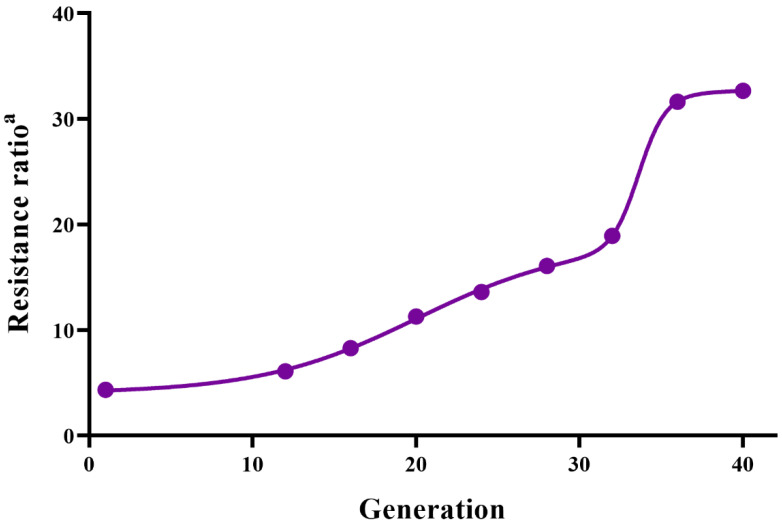
Results of resistance selection to malathion in *S. miscanthi*. ^a^ Resistance ratio = LC_50_ of the MRS strain/LC_50_ of the MSS strain.

The malathion susceptible strain (MSS) was established from original *S. miscanthi* strain through backward selections for four generations, with the ratio of susceptibility decreased by 2.7 times and the values of LC_50_ declined from 12.355 mg/L to 2.856 mg/L. The malathion resistance strain (MRS) was obtained by the continual-generation selection with malathion for 40 generations, with 32.7-fold resistance ratio compared with the MSS (Figure 1). From the trend of resistance development, the resistant ratio increased sharply from 18.92-fold for F_32_ to 31.64-fold for F_36_. The relationship between selection generations and resistance ratios of MRS was simulated and the obtained regression equation was $y=10.619e^{0.0541X}$, *R^2^* = 0.9733 and the realized heritability of *S. miscanthi* resistance to malathion was 0.02 (Table 1).

### 3.2. Cross-Resistance Pattern

The MRS strain developed 13.5-fold, 4.8-fold and 2.9-fold cross-resistance to omethoate, beta-cypermethrin and methomyl, respectively. However, cross-resistance to imidacloprid was not observed (Table 2).

### 3.3. Synergism

The effects of synergists on malathion toxicity in the MRS and MSS strains are shown in Table 3. TPP and DEF exhibited significant synergism in the MRS strain with 6.8-fold and 3.9-fold, respectively; however, no synergism was observed in the MSS strain. TPP significantly improved the susceptibility to malathion in the MRS strain, with resistance ratio decreasing from 33-fold to 3-fold. However, there were no synergistic effects for PBO and DEM in either the MRS strain or the MSS strain.

### 3.4. Biochemical Assays

The carboxylesterase activity with α-naphthyl acetate (α-NA) as substrate was higher in the MRS strain than in the MSS strain, with a 3.51-fold difference. The Michaelis constant (*K*m) in the MRS strain was reduced by half compared with the MSS strain. The maximal velocities (*V*max) of CarE were higher in the MRS strain than in the MSS strain, with differences of 2.32-fold (Table 4). Compared with the MSS strain, the activities of GSTs and AChE were higher in the MRS strain (Table 5).

## 4. Discussion

The development of *S. miscanthi* resistance to malathion showed an *S*-shaped curve with the selective process from F_0_ to F_40_. The realized heritability of malathion resistance was 0.02 in the MRS strain, indicating that for this aphid to develop 100-fold resistance to malathion, 100 successive generations are required. The development of *S. miscanthi* resistance to malathion was slow compared to *Aphis gossypi*. Only 36 generations of continuous selection were required for cotton aphid to develop 100-fold resistance to malathion [25]. This phenomenon might be related to the wide host range of cotton aphid. *S. miscanthi* has 18–20 generations per year in wheat producing areas of China, so it is difficult for *S. miscanthi* to develop high resistance. Our results are consistent with the development of *S. miscanthi* resistance to neonicotinoid insecticides in China [26].

The MRS exhibited a 13.5-fold cross-resistance to omethoate. The cross resistance may be related to both malathion and omethoate which are anti-acetylcholinesterase agents, and cause the death of insect pests by inhibiting the activity of acetylcholinesterase (AChE). The MRS strain showed 2.9-fold cross- resistance to methomyl, which is a carbamate with the same molecular target as organophosphorus insecticides. This 2.9-fold cross-resistance to methomyl might be related to the similar function of inhibiting acetylcholinesterase with organophosphorus insecticides. In addition, the MRS strain exhibited a 4.79-fold cross-resistance to beta-cypermethrin. This could be attributed to the fact that beta-cypermethrin and malathion possess the same carboxylic ester bond.

Compared with the MSS strain, both DEF and TPP showed significant synergistic effect in the MRS strain. It suggests that CarE might play an important role in *S. miscanthi* resistance to malathion by inhibition of DEF and TPP to carboxylesterase activity. This is consistent with the results of CarE activity assays. The previous study exhibited that TPP could inhibit CarE activity in *S. miscanth* with median inhibition concentrations (I_50_) of 10.55 ± 10.31 mM TPP [27]. The decrease in *K*m in the MRS strain suggested that the affinity of CarE to the substrate in MRS was more than in MSS. The assay on *V*max of CarE suggested more potential for the hydrolysis of substrate in MRS, perhaps more hydrolysis to malathion in MRS than in MSS. Another study on *Schizaphis graminum* showed that esterase activity was 2.4-fold higher in an organophosphorus-resistant strain with a-naphthyl acetate (α-NA) as the substrate [28]. Furthermore, the research on the B-biotype of *Bemisia tabaci* (Hemiptera: Aleyrodidae) demonstrated that carboxylesterase gene expression-level increased 4-fold in the organophosphorus-resistant strain [29]. Similar relative transcription levels and gene copy numbers of the CarE has been observed in the malathion resistant *A. gossypii* strain [25]. In *Bactrocera dorsalis* (Hendel) populations, constitutive and insecticide-inducible overexpression of carboxylesterases are responsible for increased detoxification of malathion [30]. In *M. persicae*, the recombinant CarE *E4* gene could hydrolyze malathion within 1.25 h by 80% [31].

Furthermore, we found that the activity of GSTs increased significantly in the MRS strain. The increase in GSTs activity was observed in many insect pests with malathion resistance. In *Bactrocera dorsalis* (Hendel), the activities of GSTs increased 3.2-fold compared to the susceptible strain [32]. The activity of GSTs was significantly greater in the resistant strain of *Lygus lineolaris* (Palisot de Beauvois) to malathion than in the susceptible strain [33]. In addition, compared with the susceptible strain, the malathion resistant strain of *Nilaparvata lugens* exhibited 1.5-fold higher in the activity of GSTs [34].

It is generally recognized that the CYP enzymes play important roles in insecticide resistance, which detoxify and activate xenobiotics at the same time. Wang et al. found that the resistant level of *Bactrocera dorsalis* to malathion was closely associated with the increase in CYP activities [32]. However, there was no significant synergism of PBO for the resistant strain of in *S. miscanth* to malathion.

Acetylcholinesterase (AChE) is a critical enzyme involved in the process of nerve transmission at the postsynaptic membrane by hydrolyzing acetylcholine released from the presynaptic terminal. The insensitivity of AChE was an important mechanism in the resistant strains of *M. persicae* and *A. gossypii* to organophosphorus insecticides [35,36].

In this study, the activity of AChE in the MRS strain was 1.73-fold higher than the MSS strain. Zhu et al. also reported that the activity of AChE was 3.3-fold higher in the organophosphorus-resistant strain than the susceptible strain. In addition, MRS exhibited 2.9-fold of low cross-resistance to methomyl, indicating that AChE might be involved in the resistance of the MRS strain to anti-acetlcholiesterase agents.

In conclusion, CarE appears to be a main mechanism of *S. miscanth* resistance to malathion. The variation in GSTs and AChE might partly contribute to *S. miscanth* resistance to malathion. These results are useful for the insecticide resistance management of wheat aphids.

## Figures and Tables

**Table 1:** Estimation of realized heritability (*h^2^*) of malathion resistance in *S. miscanthi*.

Number of Generations for Selection	Estimate of Mean Response per Generation			Estimate of Mean Selection Differential per Generation						*h* ^2^
*n*	Initial LC_50_ (Log)	Final LC_50_ (Log)	*R*	*p*	*i*	Initial Slope	Final Slope	*δ_p_*	*S*	
40	1.09	1.97	0.02	22.2	1.34	1.70	1.38	0.65	0.87	0.02

**Table 2:** Responses to other insecticides in malathion-selected population of *S. miscanthi* ^1^.

Insecticides	Strains	Slop ± SE	LC_50_ mg L^−1^ (95% Confidence Limit)	Resistance Ratio ^2^
malathion	MRS	1.383 ± 0.204	90.373 (66.752–141.708)	33.0
	MSS	1.965 ± 0.249	2.743 (2.240–3.283)	
omethoate	MRS	1.259 ± 0.199	67.786 (48.908–112.036)	13.5
	MSS	1.737 ± 0.221	5.004 (3.943–6.360)	
methomyl	MRS	1.821 ± 0.228	4.875 (3.840–6.155)	2.9
	MSS	1.887 ± 0.219	1.680 (1.339–2.068)	
beta-cypermethrin	MRS	1.781 ± 0.223	64.910 (51.452–83.560)	4.8
	MSS	2.889 ± 0.486	13.564 (10.846–16.186)	
imidacloprid	MRS	1.421 ± 0.212	15.710 (11.858–22.360)	1.6
	MSS	1.639 ± 0.213	9.786 (7.654–12.559)	

^1^ *p*  >  0.05. ^2^ RR (Resistance ratio) = LC_50_ of the MRS strain/LC_50_ of the MSS strain.

**Table 3:** Synergism of PBO, DEF, DEM and TPP on malathion in the MRS and MSS strains ^1^.

Strain	Compound	Slope ± SE	LC_50_ mg L^−1^ (95% Confidence Limit)	SR ^2^	SRR ^3^
MSS	malathion	1.97 ± 0.25	2.74 (2.24–3.28)	1	1
	+PBO	1.84 ± 0.22	2.71 (2.13–3.35)	1.01	1
	+DEF	2.05 ± 0.24	2.11 (1.66–2.57)	1.30	1
	+DEM	2.06 ± 0.23	2.42 (1.93–2.94)	1.14	1
	+TPP	1.55 ± 0.21	1.66 (0.54–2.81)	1.65	1
MRS	malathion	1.38 ± 0.20	90.37 (66.75–141.71)	1	1
	+PBO	1.47 ± 0.20	71.49 (54.94–102.26)	1.26	1.25
	+DEF	1.68 ± 0.20	17.65 (14.24–22.58)	5.12	3.94
	+DEM	1.59 ± 0.21	52.43 (41.75–68.91)	1.72	1.51
	+TPP	2.43 ± 0.243	8.09 (6.79–9.52)	11.17	6.77

^1^*p* > 0.05. ^2^ SR (synergism ratio) = LC_50_ of malathion/LC_50_ of PBO or DEF or DEM or TPP + malathion. ^3^ SRR (synergism resistance ratio) = SR of resistance strain/SR of susceptible strain.

**Table 4:** Carboxylesterase activity and kinetic parameters in *S. miscanthi*.

Strains	CarE Activity (nmol mg Protein^−1^ min^−1^)	*K*m (M)	*V*max (μmol mg Protein^−1^ min^−1^)
MSS	66.57 ± 2.80 a	4.46 × 10^−4^ ± 1.87 × 10^−4^ a	1.81 × 10^−1^ ± 2.74 × 10^−2^ a
MRS	233.56 ± 11.89 b	2.35 × 10^−4^ ± 6.28 × 10^−5^ b	4.47 × 10^−1^ ± 3.66 × 10^−2^ b

Different letters in each column indicate statistical difference by the Duncan’s Multiple Comparison test (*p* < 0.05).

**Table 5:** Glutathione *S*-transferase and acetylcholinesterase activities of the MRS and MSS strain ^1^.

Strains	GSTs Activity (nmol mg Protein^−1^ min^−1^)	AChE Activity (pmol mg Protein^−1^ min^−1^)
MSS	677.85 ± 58.64 a	42.73 ± 1.88 a
MRS	1175.78 ± 38.72 b	75.04 ± 5.86 b

^1^ Different letters in each column indicate statistical difference by the Duncan’s Multiple Comparison test (*p* < 0.05).

## Data Availability

The data that support the findings of this study are available from the corresponding author upon reasonable request.
